# Human papillomavirus infection affects the immune microenvironment and antigen presentation in penile cancer

**DOI:** 10.3389/fonc.2024.1463445

**Published:** 2024-10-18

**Authors:** Sulayne Janayna Araujo Guimarães, André Alvares Marques Vale, Mirtes Castelo Branco Rocha, Ana Luiza de Araújo Butarelli, Jenilson Mota da Silva, Amanda Jordão Silva de Deus, Leudivan Nogueira, Ronald Wagner Pereira Coelho, Silma Regina Pereira, Ana Paula Silva Azevedo-Santos

**Affiliations:** ^1^ Postgraduate Program in Health Science, Federal University of Maranhão, São Luís, Brazil; ^2^ Laboratory of Immunology Applied to Cancer, Department of Physiological Sciences, Biological and Health Sciences Center, Federal University of Maranhão, São Luís, MA, Brazil; ^3^ Laboratory of Genetics and Molecular Biology, Department of Biology, Biological and Health Sciences Center, Federal University of Maranhão, São Luís, MA, Brazil; ^4^ Maranhense Institute of Oncology Aldenora Bello, São Luís, Brazil

**Keywords:** urological carcinoma, cancer immunomodulation, dendritic cells, HPV-related cancer, costimulatory molecules

## Abstract

Penile squamous cell carcinoma (PSCC) is a largely neglected condition, predominantly affecting underdeveloped regions, and is associated with risk factors such as low socioeconomic status, phimosis, and human papillomavirus (HPV) infection. Unlike other urogenital cancers, its pathophysiology and therapeutic targets remain poorly understood, particularly regarding the immune response to the tumor microenvironment. This study aims to investigate immune cell infiltration profiles, dendritic cell maturation, and lymphocyte apoptosis in both HPV-positive and HPV-negative PSCC. Clinical and histopathological data, along with peripheral blood and tumor tissue samples, were collected from 30 patients (66.6% were HPV-positive and 33.3% HPV-negative), with an additional 19 healthy donors serving as controls. Tumor-infiltrating immune cells were analyzed following enzymatic digestion of tumor tissue, enabling detailed phenotypic characterization. A simulated tumor microenvironment was created using supernatants derived from primary cultures of HPV-positive PSCC tumors. Peripheral blood mononuclear cells were isolated and differentiated into dendritic cells (Mo-DCs) for further phenotyping and lymphoproliferation assays. Lymphocytes from healthy donors and patients were exposed to tumor culture supernatants to evaluate apoptosis induced by the tumor microenvironment. Results showed that HPV-positive tumors exhibited lower T lymphocyte frequencies compared to HPV-negative tumors. Additionally, patients infected with high-risk HPV demonstrated reduced maturation rates of Mo-DCs and decreased expression of co-stimulatory molecules on these cells compared to healthy donors. Furthermore, Mo-DCs from hrHPV-positive patients showed impaired lymphoproliferation capacity relative to controls, while HPV-negative patients exhibited a trend towards reduced lymphoproliferative ability. Regarding the simulated tumor microenvironment, lymphocytes from healthy donors underwent apoptosis, contrasting with patients' lymphocytes, which showed increased viability when cultured with tumor supernatants. These results underscore the impact of HPV infection on T lymphocyte infiltration, Mo-DC maturation, and lymphocyte survival in PSCC, offering critical insights for advancing our understanding of the tumor microenvironment and guiding the development of immunotherapy strategies.

## 1 Introduction

Penile squamous cell carcinoma (PSCC) is a rare disease in developed countries, however, it is more common in underdeveloped and developing countries (1). The Brazilian northeast region has the highest incidence of penile cancer (6,1/100,000 inhabitants) (2). Clinically, these patients are frequently characterized by late diagnosis in stage T2, grade II or III, with detection of HPV-DNA and usually undergo penectomy (2, 3). Therapy is complex and multi-layered. As it is a multifactorial disease, risk factors include phimosis, chronic inflammation, poor penile hygiene, smoking, immunosuppression, and human papillomavirus infection (1), in addition to the conditions of poverty and low access to health services, delaying diagnosis (2).

The mechanisms of carcinogenesis in PSCC have been described as HPV-dependent, through the integration of viral DNA into the host cell genome causing the overexpression of viral oncoproteins E6 and E7, host cell cycle deregulation and genomic instability of the host (3–7). Another mechanism is non-HPV related, defined by chronic inflammation and somatic genetic changes by reactive oxygen and reactive nitrogen intermediates associated with phimosis and lichen sclerosus (8–10). In both conditions, the immune system plays a fundamental role in the establishment and maintenance of the tumor microenvironment.

The dynamic interaction of neoplastic, immune, stromal, and vascular cells determines the survival of malignant cells in tissues and organs (11, 12). Chu et al. (13) reported immunophenotype similarities between PSCC HPV-positive and HPV-negative patients. However, the HPV-positive tumors showed higher density of intratumoral PD-1 (13). Furthermore, HPV infection reduces inflammation-associated signaling by decreasing the destruction of infected cells (14) and promoting the production of viral proteins E6 and E7 from high-risk HPV strains. These proteins inhibit key immune responses, suppressing interferon signaling pathways (15) and E7 downregulating TLR9 expression in phagocytes (16). This suppression impairs the mechanisms necessary for effective immune recognition and response, allowing the virus to evade the host’s immune system.

The HPV infection and the host immune response are integral components of the tumor microenvironment in PSCC and the Dendritic Cells (DCs) can be an interesting target as they orchestrate the immune response, serving as the primary antigen-presenting cells that determine lymphocyte activation and the profile of the adaptive immune response, whether effector or tolerogenic (17). So far, the studies on immune aspects associated with penile cancer have been based on theoretical approaches. This work proposes an experimental study using immune cells from penile cancer patients, evaluating the impact of HPV on the process of antigen activation and presentation, identifying mechanisms of immune evasion, and targets for immunotherapeutic strategies.

## 2 Materials and methods

### 2.1 Patient and sample collection

Patients’ blood and PSCC tissue samples were collected at Aldenora Bello Cancer Hospital, São Luis-Brazil, from 2017 to 2020. This study was approved by the Research Ethics Committee on Humans from the Federal University of Maranhão and by the National Research Ethics Commission (CONEP‐ Brazil, CAAE: 46371515.5.0000.5087).

### 2.2 Study design

This study employed experimental research techniques utilizing both *in vitro* and *ex vivo* methodologies. Peripheral blood was collected from patients (n=30) and healthy donor volunteers (n=19). The tumor tissue from patients was enzymatically digested to characterize the immune infiltrating cells and to establish *ex vivo* cultures. HPV genotyping was performed using nested-PCR, following the protocol provided by BioGenetics Molecular Technologies (Uberlândia, Minas Gerais, Brazil; patent number BR102017004615.0), to identify viral subtypes classified as high risk (16, 18, 31, 33, 35, 39, 45, 51, 52, 56, 58, 59, 66, 68, 73, and 82), intermediate risk (MM7, MM8, 26, 30, 34, 53, 54, 55, 61, 62, 64, 67, 69, 70, 71, 72, 74, and 81), and low risk (6, 11, 42, 43, 44, and 57). In addition, we performed DNA sequencing according to our previous protocol (3). The tumor cell culture supernatants were used as tumor microenvironment-like to perform the apoptosis lymphocyte assay. From the peripheral blood mononuclear cells (PBMCs), the monocytes were isolated for *in vitro* differentiation in mature Dendritic Cells (Mo-DCs) and the lymphocytes were used in proliferation and apoptosis assay. Mo-DCs activation was evaluated through the expression of costimulatory molecules and compared with patients’ groups and healthy donors (**Supplementary Figure 1**).

### 2.3 Analysis of tumor-infiltrating immune cells

Tumor tissues fragments were treated with Collagenase IV (0.056 mg/mL of RPMI 1640 medium) according to the manufacturer (Sigma-Aldrich, Saint Louis,USA) at 37°C for 2 hours (18) After enzymatic digestion, the cells were stained with 0.04% Trypan Blue dye and counted using a Neubauer chamber. Phenotypic analysis by flow cytometer was performed on 2x10^5^ tumor cells, as described below. Additionally, 1x10^4^ tumor cells were cultured in 12-well plates for 5 days. After 5 days, the supernatant was removed from the plate and centrifuged to remove non-adherent cells that remain in suspension in the medium. For the apoptosis assay, the tumor supernatant was added to the lymphocyte culture at a concentration of 10%. This assay was conducted over three days in 96-wells plates containing 100 µL of supplemented medium.

### 2.4 *In vitro* differentiation of Mo-DCs

Lymphocyte activation depends on the antigen presentation mechanism by Antigen-Presenting Cells (APCs), mainly represented by DCs. Thus, monocytes obtained from the peripheral blood of patients with HPV-positive and HPV-negative PSCCa were differentiated *in vitro* into dendritic cells. Buffy coat of peripheral blood (5-10mL) samples from patients and healthy blood donors were used for the isolation of peripheral blood mononuclear cells (PBMCs) and plasm. Dendritic Cells from both groups were produced by standard procedures (18). Briefly, PBMCs were isolated from peripheral blood buffy coats using Ficoll density gradient. After washing with phosphate buffered saline, 1x10^6^ cells/mL were plated and kept in culture with cell culture medium (RPMI 1640, Gibco, New York, USA) supplemented with 10% of fetal bovine serum (FBS), 1% of Antibiotic-antimycotic (Gibco, New York, USA) overnight, to separate adherent cells (monocytes) from non-adherent cells (lymphocytes). The lymphocytes were separated for future proliferation and apoptosis assays. The monocytes were seeded in 12-well plates and cultured in RPMI 1640 medium with 10% FBS added 50 ng/mL^−1^ recombinant human GM-CSF (PeproTech, Cranbury, USA), and 50 ng/mL^−1^ IL-4 (PeproTech, Cranbury, USA) to generate immature DCs. On the fifth day of culture, 20 ng/mL^−1^ TNF-α (PeproTech, Cranbury, USA) was added to the culture to generate mature monocyte-derived dendritic cells (Mo-DCs). After 7 days of culture, the cells were harvested, washed once with PBS, and used for the experiments.

### 2.5 Immune cells phenotype

A total of 1x10^5^ cells were incubated, at room temperature, for 30 min, with PBS and antibody: CD14 (BD Biosciences Cat# 555397, RRID: AB_395798), CD86 (BioLegend Cat# 305422, RRID: AB_2074981), HLA-DR (BD Biosciences Cat# 339194, RRID: AB_647443), CD3 (BioLegend Cat# 300312, RRID: AB_314048), CD8 (BioLegend Cat# 300906, RRID: AB_314110), CD4 (BD Biosciences Cat# 555347, RRID: AB_395752), CD19 (BioLegend Cat# 302210, RRID: AB_314240), and CD56 (BD Biosciences Cat# 555516, RRID: AB_395906) (**Supplementary Table 1**). Following incubation, cells were washed with PBS, and analyzed using an InCyte Guava flow cytometer (Luminex Corporation). Data analysis was performed using FlowJo software (v.10; RRID: SCR_008520).

### 2.6 Allogeneic lymphocytes proliferation index

To assess the ability of Mo-DCs to induce lymphocyte activation, the allogeneic lymphocytes obtained from healthy donors were labeled with 2.5 μM carboxyfluorescein succinimidyl ester (CFSE) (Sigma-Aldrich,Saint Louis, USA) and co-cultured with patients’ or healthy donors’ Mo-DCs for 3 days. Fluorescent signals were analyzed using a flow cytometer, following the manufacturer’s instructions. A minimum of 10,000 events were acquired using a FACSCalibur flow cytometer and analyzed with cytometer software (FlowJo Software, v.10; RRID: SCR_008520).

### 2.7 Analysis of lymphocyte apoptosis

To simulate the effect of the tumor microenvironment on lymphocyte viability, the *ex vivo* culture supernatant from the tumor tissue was used as treatment. The healthy donor and patient’s lymphocytes were incubated for three days in a 96-well plate containing 100 µL of RPMI medium supplemented with 10% tumor supernatant. Fluorescence-conjugated Annexin V and propidium iodide were utilized for apoptosis analysis (BD Pharmingen, Franklin Lakes, USA) following the manufacturer’s instructions. After the incubation, the lymphocytes isolated from patients and healthy donors were washed and incubated in Annexin V binding buffer at a 1:20 dilution for 15 minutes at room temperature. Subsequently, the cells were then washed, resuspended in a fresh Annexin V binding buffer, and promptly analyzed by flow cytometry, according to the manufacturer’s guidelines.

### 2.8 Statistical analysis

Statistical analyses were performed using GraphPad Prism software (v10). Data are presented as the mean ± standard error of the mean (SEM). The Shapiro-Wilk test was used to assess the normality of data distribution. Depending on the distribution (normal or non-normal), appropriate parametric or non-parametric tests were applied. For comparisons between two groups, the parametric t-test or the non-parametric Mann-Whitney U test (both two-tailed and unpaired) were used. For comparisons involving three or more groups, either the parametric one-way ANOVA followed by Tukey’s multiple comparison *post hoc* test or the non-parametric Kruskal-Wallis test followed by Dunn’s multiple comparison *post hoc* test was employed. A p-value < 0.05 was considered statistically significant, with significance levels indicated as *p < 0.05, **p < 0.005, and ***p < 0.0005. The sample size for each test and the specific test used in each analysis are detailed in the figure legends.

## 3 Results

### 3.1 Penile cancer patients profile

Most of the patients enrolled in the study have low levels of education. The main occupation was agriculture, and the majority were married. The average age of the patients was 60.18 (26-94 year) years old, and reported smoking and drinking habits (50% and 53.3%, respectively). Phimosis was present in 40%, but this information was not available for 40% of the patients. The most frequent site of the tumor was the glans (46.6%) or the glans associated with other areas (39.9%).

All tumors were squamous cell carcinoma with 56.5% HPV-related (Warty carcinoma: 33.3%; Basaloid: 6.6%; Warty-basaloid: 16.6), 26.6% non-HPV related, and 16.6% had no information. Histological grades I (16.6%) and II (16.6%) were the most frequent, however, this information was not available for 40% of tumors. The size of the tumor for most patients (70%) was 0.7 to 5.4 cm, and the tumors were defined as pT1 (13.3%), pT2 (20%), pT3 (23.3%), and pT4 (3.3%). However, 36.6% of this information was not available in the medical records. All tumor samples were tested for HPV DNA detection using nested-PCR, with 66.6% testing positive. Among the HPV-positive samples used in immunological assays, HPV16 was the most common genotype, present in 66.7% of cases, including one case of co-infection with HPV6. Additionally, 33.3% of the samples were positive for HPV18, defined as high-risk oncogenic HPV (hrHPV). **Table 1** provides an overview of the sociodemographic, clinical, and pathological characteristics of the patients.

**Table 1:** Penile cancer socioeconomic, clinical, and pathologic profile.

Socioeconomic features		No. Cases (%)
Formal education	No education	15 (50)
	Primary education	13 (43.3)
	Secondary education	0
	Tertiary education	0
	No information	2 (6.6)
Occupation	Farm hand	19 (63.3)
	Fisherman	1 (3.3)
	Retired	6 (20)
	Others	2 (6.6)
	No information	2 (6.6)
Marital status	Single	6 (20)
	Married/Stable union	14 (46.6)
	Divorced	3 (10)
	Widower	4 (13.3)
	No information	3 (10)
Pathologic features		No. Cases (%)
Anatomical site	Glans	14 (46.6)
	Foreskin	2 (6.6)
	Glans + foreskin	5 (16.6)
	Glans and other areas	7 (23.3)
	No information	2 (6.6)
Histological subtypes	Non-HPV related/SCC usual type	8 (26.6)
	HPV related/Warty carcinoma	10 (33.3)
	HPV related/Basaloid SCC	2 (6.6)
	HPV related/Warty–basaloid carcinoma	5 (16.6)
	No information	5 (16.6)
Pathologic features		No. Cases (%)
Anatomical site	Glans	14 (46.6)
	Foreskin	2 (6.6)
	Glans + foreskin	5 (16.6)
	Glans and other areas	7 (23.3)
	No information	2 (6.6)
Histological subtypes	Non-HPV related/SCC usual type	8 (26.6)
	HPV related/Warty carcinoma	10 (33.3)
	HPV related/Basaloid SCC	2 (6.6)
	HPV related/Warty–basaloid carcinoma	5 (16.6)
	No information	5 (16.6)
Histological grade	Grade 1	5 (16.6)
	Grade 2	5 (16.6)
	Grade 3	6 (20)
	Grade 4	2 (6.6)
	No information	12 (40)
Tumor size (cm)	0,7-5,4	21 (70)
	5,4-10,1	2 (6.6)
	14,8-19,5	1 (3.3)
	No information	6 (20)
Tumor staging	pT1	4 (13.3)
	pT2	6 (20)
	pT3	7 (23.3)
	pT4	1 (3.3)
	pTi	1 (3.3)
	No information	11 (36.6)
HPV status	Positive	20 (66.6)
	Negative	9 (30)
	No information	1 (3.3)

### 3.2 Immune-infiltrated cells in hrHPV-positive and HPV-negative PSCC

**Figure 1:**
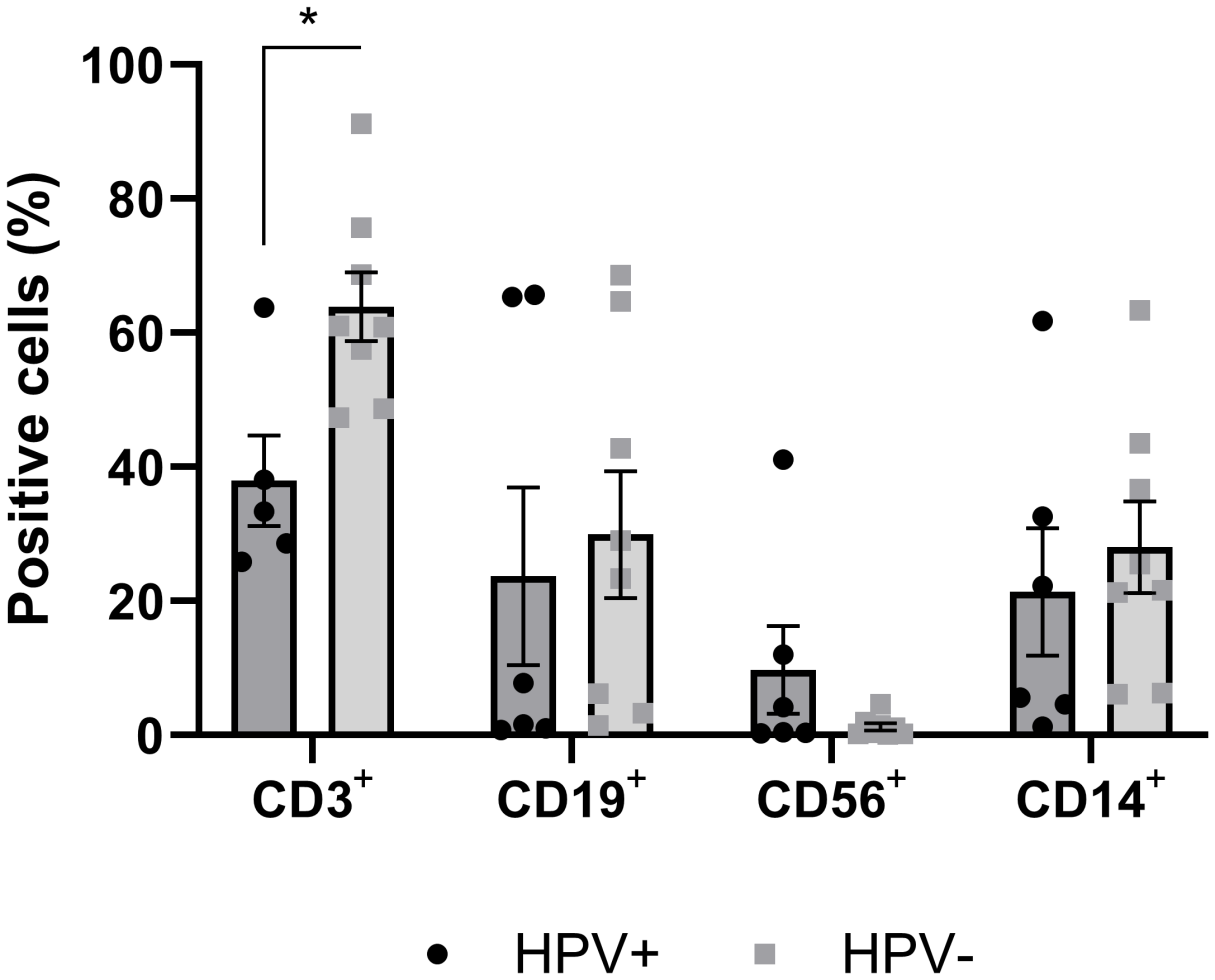
Frequency of cells positive for immune cell markers infiltrated in tumor tissue of hrHPV-positive and HPV-negative PSCC patients. Tumor tissue samples were enzymatically digested and labeled with surface markers CD3+, CD19+, CD56+, and CD14+, to identify T lymphocytes, B lymphocytes, Natural Killer (NK) cells, and Monocytes/Macrophages, respectively. Data were presented as mean ± standard error of the mean (SEM), and an unpaired t-test was performed for each marker, with statistical significance set at *p<0.05, comparing HPV-positive (n=8) and HPV-negative (n=8) carcinoma samples.

The expression of specific markers CD3+ for T lymphocytes, CD19+ for B lymphocytes, CD56+ for NK cells, and CD14+ for monocytes/macrophages was used to assess immune cell infiltration within the tumor. The results revealed that the frequency of T lymphocytes was significantly lower in hrHPV-positive (37.96 ± 6.78) compared to HPV-negative PSCC (63.89 ± 5.12, p=0.0104). An inverse trend was observed in the frequency of NK cells, with a higher frequency in hrHPV-positive (3.44 ± 2.26) compared to HPV-negative cases (1.27 ± 0.54, p=0.2698). No significant differences (p≥0.05) were observed in the average frequencies of B lymphocytes and monocytes/macrophages between the two groups (**Figure 1**).

### 3.3 Dendritic cells phenotype and function associated to hrHPV-positive and HPV-negative PSCC

**Figure 2:**
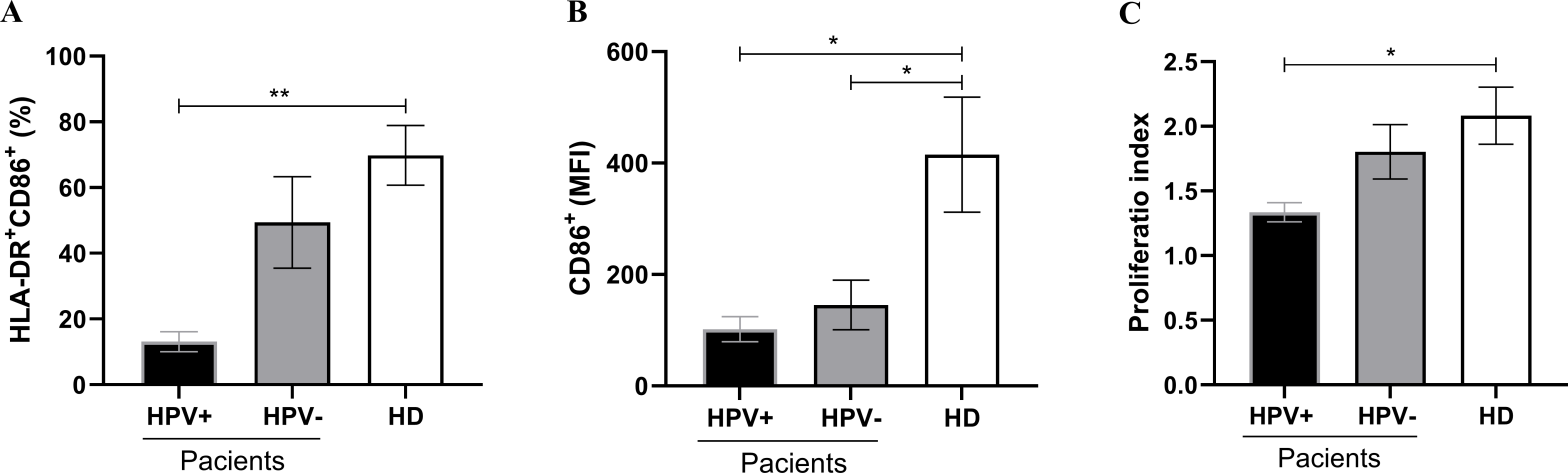
Comparison of the phenotypic and functional profile of Mo-DCs obtained from patients with hrHPV-positive and HPV-negative PSCC patients, compared to healthy donors. **(A)** Frequency of Mo-DCs expressing the co-stimulatory molecule CD86 in patients with hrHPV-positive (n=6) and HPV-negative (n=6) PSCC, compared to healthy donors (n=9). **(B)** Mean Fluorescence Intensity (MFI) of CD86 on positive cells from patients with hrHPV-positive (n=6) and HPV-negative (n=6) PSCC, compared to healthy donors (n=9). **(C)** Proliferation index of healthy allogeneic lymphocytes co-cultured with Mo-DCs derived from hrHPV-positive (n=6) and HPV-negative (n=6) PSCC patients, compared to healthy donors (n=6). Data are presented as mean ± standard error of the mean (SEM). Statistical analysis was performed using One-way ANOVA followed by Tukey’s multiple comparison post hoc test, with significance set at p<0.05. Significance levels are denoted as *p < 0.05 and **p < 0.01.

Comparing the frequency of Mo-DCs positive for CD86 between patients and healthy donors, it was observed that there was a significant reduction in cells obtained from hrHPV-positive patients (13.10 ± 3.03; healthy donors: 69.81 ± 9.10, p=0.0025). However, despite a trend towards reduction, the frequency was not significant in HPV-negative patients (49.36 ± 13.92, p= 0.3180). As for the frequency of Mo-DCs expressing co-stimulatory molecules, the results showed that hrHPV-positive patients have a lower proportion of positive cells compared to cells obtained from HPV-negative patients, but without significance (**Figure 2A**). The intensity of CD86 molecule expression on the membrane of Mo-DCs was lower in PSCC patients, both hrHPV-positive (101.8 ± 22.30; p=0.0293) and HPV-negative (145.2 ± 44.68; p=0.0490), compared to healthy donors (415.0 ± 103.4) (**Figure 2B**). The ability of Mo-DCs to induce lymphocyte proliferation was lower in cells from hrHPV-positive patients compared to healthy donors (Proliferation Index respectively: 1.33 ± 0.07; 2.08 ± 0.22, p=0.0275). No difference was observed in the proliferation index between HPV-negative patients and healthy donors. There was also no significant difference in the ability to induce lymphoproliferation between patient groups; however, the data showed a trend towards reduction in cells obtained from hrHPV-positive patients compared to HPV-negative patients (Proliferation Index respectively: 1.33 ± 0.07; 1.80 ± 0.21, p=0.1668) (**Figure 2C**). Interestingly, these findings correlate with the CD86 expression and frequency of positive cells.

### 3.4 Lymphocyte viability in the presence of primary culture supernatant from PSCC

**Figure 3:**
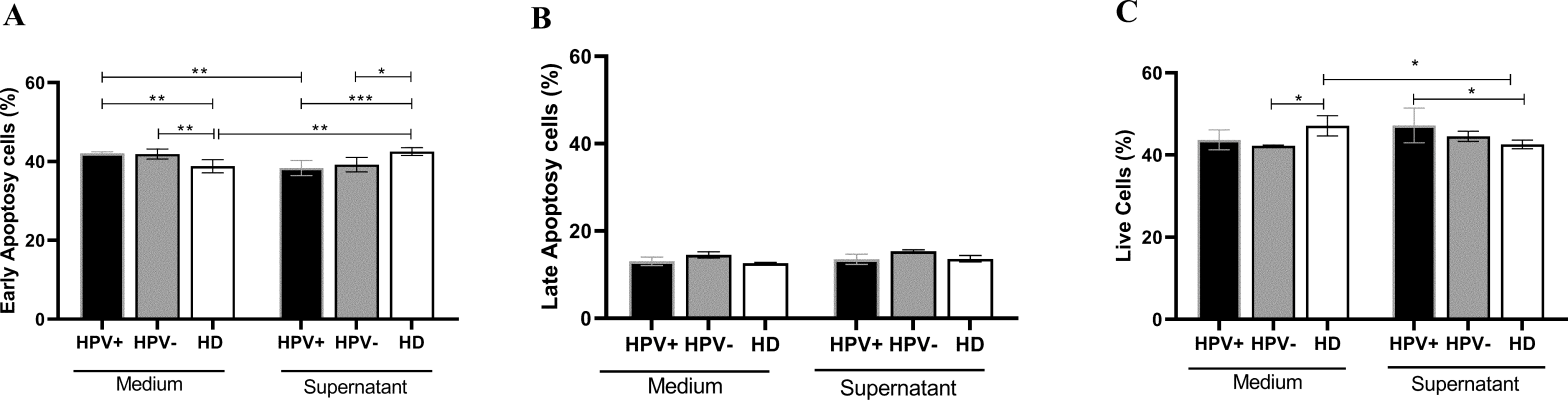
Viability of lymphocytes obtained from PSCC patients with hrHPV-positive and HPV-negative carcinoma and healthy donors, treated with supernatant obtained from *ex vivo* culture of tumor cells from penile carcinoma. **(A)** Frequency of lymphocytes in early apoptosis from patients with hrHPV-positive (n=6) and HPV-negative (n=6) PSCC, compared to healthy donors (n=5). **(B)** Frequency of lymphocytes in late apoptosis from hrHPV-positive (n=6) and HPV-negative (n=6) PSCC patients, compared to healthy donors (n=5). **(C)** Frequency of viable lymphocytes from hrHPV-positive (n=6) and HPV-negative (n=6) PSCC, compared to healthy donors (n=5). Data are presented as the mean ± standard error of the mean (SEM). Statistical analysis was conducted using One-way ANOVA followed by Tukey’s multiple comparison post hoc test and an unpaired t-test, with significance set at p<0.05. Statistical significance is denoted as *p < 0.05, **p < 0.01, and ***p < 0.001.

Assessing the impact of the tumor microenvironment on the induction of apoptosis in lymphocytes, the results showed that lymphocytes from healthy donors (38,80 ± 0,6938) exhibited a lower initial apoptosis rate compared to lymphocytes from hrHPV-positive (42,07 ± 0,1687, p=0,0067) and HPV-negative (41,90 ± 0,6351, p=0,0229) patients when cultured in medium. However, when lymphocytes were cultured in the presence of tumor supernatant, lymphocytes from healthy donors showed a significant increase in apoptotic cells (42.53 ± 0.40, p= 0.0018), compared with healthy donors. Interestingly, lymphocytes from hrHPV-positive patients had a reduction in the frequency of apoptotic cells when cultured with tumor supernatant (38.37 ± 0.78), when compared to culture with medium only (42.07 ± 0.16, p=0.0019) (**Figure 3A**). The data remained consistent across groups in different treatments when evaluating late apoptosis (**Figure 3B**). On the other hand, the frequency of viable cells was higher in lymphocytes from healthy donors (47.10 ± 1.00) cultured in the medium compared to hrHPV-positive (43.70 ± 0.98, p=0.0392) and HPV-negative patients (42.20 ± 0.11, p=0.0496). However, there was a reduction in viability of lymphocytes from healthy donors (42.53 ± 0.40) when treated with tumor supernatant (47.10 ± 1.00, p=0.0392) (**Figure 3C**). The data on viability and initial apoptosis complement each other, both in lymphocytes from healthy donors and patients.

## 4 Discussion

In this study, the patients exhibited socioeconomic aspects related to poverty, including low economic status, low educational attainment, and rural occupation. The northeastern region of Brazil comprises states with the lowest development index in the country, being the region with the highest incidence of this cancer (9, 19, 20). Although the incidence and mortality rates of penile squamous cell carcinoma (PSCC) are predominantly concentrated in developing regions, recent studies have documented a rising trend in the incidence within developed countries (20). The data reinforce the importance of social policies aimed at education and improving the quality of life as a way to mitigate the disease.

PSCC was the most common among the patients studied, with the glans and prepuce being the most affected anatomical sites. In a study conducted at the Netherlands Cancer Institute, 84.5% of squamous cell carcinomas did not have the histological subtype identified (21). The classification of histological subtypes is important for the diagnosis of penile carcinoma, yet it is often neglected. Furthermore, upon reviewing the medical records, it was noted that some patients did not have documented data regarding HPV status at the time of diagnosis.

HPV infection is another significant risk factor in penile carcinogenesis. PSCC includes numerous histological subtypes, some of which are associated with HPV, and it is often an aggressive tumor (22–24). The data showed that both histopathological evaluation (56.5%) and Nested-PCR (66.6%) revealed that most patients had HPV infection. Macedo et al. (3) investigated HPV genotyping in patients from the same region, revealing a 96.4% frequency, with high-risk subtypes being predominant (88.6%), representing one of the highest rates ever recorded. This finding highlights the epigenetic changes induced by HPV in penile carcinogenesis (3), reinforcing the importance of high-risk HPV subtype infections in the development of this cancer.

The results of this study highlighted the importance of viral infection in modulating the immune response in the tumor microenvironment, underscoring the need to define the virus’s role in PSCC. Moreover, the data showed that some patients who tested negative for HPV by Nested-PCR showed histopathological evidence of cellular changes (koilocytosis), suggestive of HPV-associated carcinoma subtypes. HPV was identified in 42% of patients with PSCC, applying PCR assay (25). Despite being a standard technique for HPV diagnosis, this study reinforces other works that demonstrate false-negative results for HPV in PSCC samples. Considering this context, the importance of HPV in this type of tumor may be greater than previously indicated, making vaccination a powerful alternative in combating it. Moreover, this scenario underscores the significance of using additional techniques for the accurate detection of HPV infection.

Histological and immunohistochemical studies have evaluated the presence of immune cells infiltrating penile carcinoma, but the results are controversial as to density, immune profile, and the presence or absence of HPV (11–13, 26–28). On the other hand, a systematic review demonstrates that a higher presence of immune cells infiltrating penile carcinoma is associated with greater patient survival (29). In this work, flow cytometry was applied to a cell suspension obtained from tumor tissue. The data showed the presence of a phenotypic profile with both innate and adaptive immune cells in penile carcinoma. When analyzing the presence of HPV, hrHPV-positive carcinomas showed a lower frequency of T lymphocytes, and a trend for a higher frequency of NK cells compared to HPV-negative PSCC.

Viral infection induces the activation of NK cells, which can recognize and kill virus-infected cells that have down regulated surface MHC I molecules and are resistant to cytotoxic T lymphocyte-mediated death (30). In this study, the tumor-infiltrating immune cells showed that the HPV presence can modulate the immune response to an innate profile, reducing the antigen-specific response and, consequently, a better anti-tumor immune response. Thus, the data suggest that mechanisms associated with HPV can not only contribute to carcinogenesis through the infection of the virus in the host cell but also through immunoregulatory mechanisms that shape the tumor microenvironment.

In penile cancer, the overexpression of p16INK4a was associated with the presence of hrHPV and basaloid subtype tumors (31). Moreover, studies indicate that p16INK4a expression triggers a senescence-associated secretory phenotype in the tumor microenvironment, promoting the release of inflammatory cytokines. This cytokine release inhibits the activity of cytotoxic T lymphocytes and induces immunosuppression (32).

In HPV-infected epithelium, APCs also exhibit immature phenotypes characterized by the downregulation of surface MHC molecules and co-stimulatory molecules, reducing the capacity of DCs to stimulate antigen-specific T cells (33). To the best of our knowledge, this is the first study in which dendritic cells have been differentiated from monocytes of patients diagnosed with penile carcinoma. The presence of hrHPV was significant in determining an immature profile in DCs, reducing mature-DCs presence, and the capacity to express co-stimulatory molecules, suggesting viral modulation of tolerogenic immunity. However, the formation of the tumor microenvironment independent of HPV also reduced CD86 expression, indicating that there is a failure in antigen presentation as a tumor escape mechanism, which is a target for immunotherapeutic strategies. Induction of an immature profile of dendritic cells with a lower expression of co-stimulatory molecules has already been reported as a tumor escape mechanism for gastrointestinal, lung, breast, and other cancers (34–38), and the present study reinforces the participation of HPV in the formation of the microenvironment unfavorable to maturation of DCs.

The role of lymphocytes in the antitumor response has been studied and applied in immunotherapy (39, 40). Adoptive cell therapy (ACT) with tumor-infiltrating lymphocytes (TIL) initiated with the expansion of TILs from surgically resected human tumor samples has been applied (41). In this study, it was found that T lymphocytes from healthy donors are induced to undergo apoptosis when exposed to soluble factors produced by the tumor; on the other hand, T lymphocytes from patients showed greater viability. A study comparing the presence of cytotoxic T lymphocytes among HPV-positive and HPV-negative PSCC did not observe any differences (21, 41). Conversely, in usual subtype penile carcinoma, unrelated to HPV, the presence of infiltrating regulatory T cells was associated with poor survival and unfavorable prognosis (42). Thus, understanding the resistance of these lymphocytes to the tumor microenvironment simulation can lead to a better understanding of the mechanisms of tumor escape associated with HPV.

A review of patients with hrHPV-positive cervical cancer demonstrates that viral proteins increase the expression of Cyclooxygenase-2 (COX-2), leading to chronic inflammation. This, in turn, enhances the recruitment of Myeloid-Derived Suppressor Cells (MDSCs), shifts the immune response profile from Th1 to Th2, and promotes the differentiation of M2 macrophages and regulatory T cells (Tregs) (43). Similar findings have been observed in head and neck carcinomas (44). Data from this study indicate that the tumor microenvironment supports the viability of lymphocytes obtained from patients, particularly those with hrHPV-positive PSCC patients. On the other hand, it shows that the profile of DCs in these patients presents an immature phenotype and with a lower frequency of T lymphocytes infiltrating the tumor.

Thus, the presence of HPV appears to trigger an immune response that is insufficient to eliminate the tumor, thereby contributing to the progression of penile cancer. The data presented here deepen our understanding of HPV’s role in modulating the immune response in PSCC patients. This not only elucidates the pathophysiological mechanisms associated with the infection but also identifies potential therapeutic targets for immunotherapy as a promising strategy. Moreover, the immunological implications highlighted reinforce and expand the need for HPV vaccination as a means to prevent and/or mitigate both the local and systemic impacts of penile cancer.

## Data Availability

Original contributions presented in the study are included in the article and supplementary materials. Further inquiries can be directed to the corresponding author.
