# Supplementary figures and images for: Human papillomavirus infection affects the immune microenvironment and antigen presentation in penile cancer
Source: Front Oncol. 2024 Oct 18;14:1463445. doi: 10.3389/fonc.2024.1463445 (PMC11527599; doi:10.3389/fonc.2024.1463445)

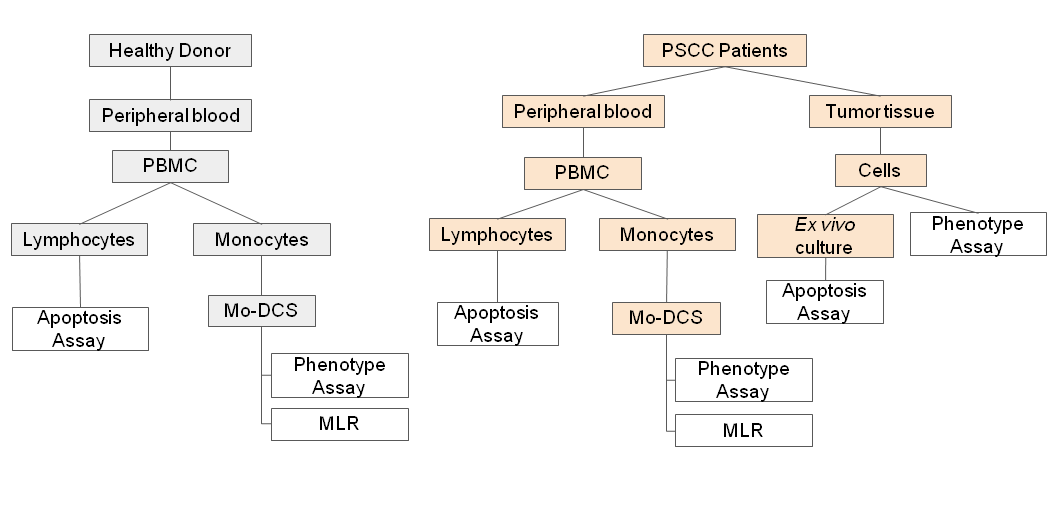

Supplement: Supplementary file 1 [file Image1.tif]
