# Supplementary material for: Human papillomavirus infection affects the immune microenvironment and antigen presentation in penile cancer
Source: Front Oncol. 2024 Oct 18;14:1463445. doi: 10.3389/fonc.2024.1463445 (PMC11527599; doi:10.3389/fonc.2024.1463445)
Supplement: Supplementary file 2 [file Table1.docx]

**Table S1.** Set of immunophenotyping assay markers.

| **Aim** | **Marker** | **Catalog** | **Clone** | **Fluorophore** | **Company** |
| --- | --- | --- | --- | --- | --- |
| **mo-DC phenotype** | **CD14** | **555397** | **--** | **FITC** | **BD** |
|  | **HLA-DR** | **339194** | **--** | **PerCP Cy 5.5** | **BD** |
|  | **CD86** | **305422** | **IT2.2** | **PE Cy7** | **Biolegend** |
| **Lymphocyte phenotype** | **CD3** | **300312** | **HIT3A** | **APC** | **Biolegend** |
|  | **CD4** | **555347** | **--** | **PE** | **BD** |
|  | **CD8** | **300906** | **HIT8A** | **FITC** | **Biolegend** |
|  |  |  |  |  |  |
| **Tumor-infiltrating immune cells phenotype** | **CD14** | **555397** | **--** | **FITC** | **BD** |
|  | **HLA-DR** | **339194** | **--** | **PerCP Cy 5.5** | **BD** |
|  | **CD86** | **305422** | **IT2.2** | **PE Cy7** | **Biolegend** |
|  | **CD3** | **300312** | **HIT3A** | **APC** | **Biolegend** |
|  | **CD14** | **555397** | **--** | **FITC** | **BD** |
|  | **CD19** | **302210** | **HIB19** | **PE Cy5** | **Biolegend** |
|  | **CD56** | **555516** | **--** | **PE** | **BD** |

CD: Cluster differentiation; FITC: fluorescein isothiocyanate; PE R-phycoerythrin; APC: Allophycocyanin.
